# Amyloid Precursor Protein Is Trafficked and Secreted via Synaptic Vesicles

**DOI:** 10.1371/journal.pone.0018754

**Published:** 2011-04-27

**Authors:** Teja W. Groemer, Cora S. Thiel, Matthew Holt, Dietmar Riedel, Yunfeng Hua, Jana Hüve, Benjamin G. Wilhelm, Jürgen Klingauf

**Affiliations:** 1 Department of Membrane Biophysics, Max Planck Institute for Biophysical Chemistry, Göttingen, Germany; 2 Institute of Medical Physics and Biophysics, University of Münster, Münster, Germany; 3 Department of Neurobiology, Max Planck Institute for Biophysical Chemistry, Göttingen, Germany; 4 Electron Microscopy Group, Max Planck Institute for Biophysical Chemistry, Göttingen, Germany; 5 Fluorescence Microscopy Facility Münster, Institute of Medical Physics and Biophysics, University of Münster, Münster, Germany; 6 Department of Psychiatry and Psychotherapy, University of Erlangen, Erlangen, Germany; Tokyo Medical and Dental University, Japan

## Abstract

A large body of evidence has implicated amyloid precursor protein (APP) and its proteolytic derivatives as key players in the physiological context of neuronal synaptogenesis and synapse maintenance, as well as in the pathology of Alzheimer's Disease (AD). Although APP processing and release are known to occur in response to neuronal stimulation, the exact mechanism by which APP reaches the neuronal surface is unclear. We now demonstrate that a small but relevant number of synaptic vesicles contain APP, which can be released during neuronal activity, and most likely represent the major exocytic pathway of APP. This novel finding leads us to propose a revised model of presynaptic APP trafficking that reconciles existing knowledge on APP with our present understanding of vesicular release and recycling.

## Introduction

Amyloid precursor protein (APP) is a type 1 membrane-spanning protein of approximately 120 kDa, which is ubiquitously expressed in mammalian cells. The protein has received particular attention because of its role in the nervous system. Under normal physiological conditions APP is involved in synapse formation and function. However, it is the involvement of this protein in the pathology of Alzheimer's disease that has raised particular interest. Alzheimer's disease is the most prevalent neurodegenerative disease facing western populations. Dysregulation of APP trafficking is thought to play a central role in the progression of this condition, because formation of the senile plaques characteristic of the disease is intimately linked to APP metabolism [1].

As such there is considerable interest in understanding both the molecular processing and trafficking pathways of this protein in neurons. Indeed over the last twenty years, these problems have formed the basis of intensive investigations by a large number of groups. Unfortunately, while the molecular events surrounding APP processing have been comprehensively elucidated, the cellular mechanisms regulating its intracellular trafficking in neurons remain unclear.

To date, the most widely accepted model of APP trafficking in the presynaptic terminal is one put forward by Cirrito and colleagues [2]. This integrates results from their own *in vivo* microdialysis experiments with knowledge of APP transport in tissue culture cells to present a model of synaptic trafficking consistent with the known molecular processing of this protein. In this model, full-length APP is constitutively transported from the ER-Golgi network to the cell surface, where cleavage by α-secretase results in the release of a 100–110 kDa soluble fragment (sAPPα), which plays a crucial role in synapse formation and maintenance [3]. However, only a fraction of APP is thought to be cleaved at the cell surface and the protein can be further processed by internalization, via clathrin-mediated endocytosis, to an early endosomal compartment [2]. Here, the molecule is cleaved by the sequential actions of beta site APP cleaving enzyme 1 (BACE 1) and the γ-secretase complex to produce a soluble N-terminal fragment (sAPPβ) and a 37–49 amino acid amyloid β-peptide (Aβ) – the so-called ‘amylogenic pathway’ [4]. Once produced, these protein fragments are trafficked back to the plasma membrane for subsequent secretion [5]. In the brain sAPPβ promotes axonal pruning via caspase activation [6]. Aβ-oligomers inhibit long-term potentiation, suggesting an important role in modulating synaptic plasticity and synaptic scaling under physiological conditions, where the levels of Aβ are controlled by regulation of both production and degradation. It is overproduction of Aβ, commonly due to mutations in APP or its processing enzymes, that is considered to act pathologically, leading to concentration-dependent formation of amyloid plaques, neurotoxicity and synapse loss [7]. The remaining amino-terminal APP intracellular domain (AICD) may serve as a transcription factor [8]. Interestingly, formation of sAPPα and sAPPβ/Aβ are thought to be mutually exclusive, as cleavage by α-secretase occurs within the BACE recognition site, allowing potential modulation of the amylogenic pathway. A more detailed description of the molecular processing of APP and its functions can be found in reviews that have recently been published [9], [10].

Unfortunately, investigating the trafficking of APP in neurons, in particular its role within the secretory apparatus, has always been complicated by two major factors. First, endogenous APP is expressed at very low levels, typically at the limit of assay sensitivity, in both rat and mouse neurons [11]. Hence the usual approach to studying APP in neurons has been based on overexpression of the protein, either acutely in culture conditions or chronically in transgenic mouse models, even though the exogenous protein may not always traffic correctly when expressed at high levels. Second, techniques such as microdialysis do not directly assess presynaptic trafficking pathways, as they only measure the terminal event (release) – explaining the reliance on data obtained from tissue culture cell models [2]. Thus, some intriguing details have remained elusive, which have hindered attempts to draw a completely integrated pathway for APP trafficking at the synapse. For instance, although endogenous full-length APP was found in clathrin-coated vesicles, which represent the main recycling pathway for synaptic vesicle recycling in brain, it was proposed that APP is sorted away from synaptic vesicle proteins during the recycling process [12]. Given that synaptic vesicle proteins are generally thought to enrich to the vesicle, the small amount of APP found in purified synaptic vesicle fractions has, therefore, generally been regarded as a contaminant - and it became accepted that APP (derivatives) are not released during exocytosis of synaptic vesicles. Thus, the exact identity of the secretory organelle remains enigmatic, despite the fact that Aβ release has many of the hallmarks of synaptic vesicle release and recycling (including sensitivity to tetrodotoxin, tetanus toxin and dynamin inhibitors) [2], [5].

Recently, however, Frykman and colleagues found that γ-secretase is highly enriched in a crude synaptic vesicle fraction, together with APP cleavage products [13]. And, much to our surprise, we also found APP in a fraction of highly pure synaptic vesicles during a routine proteomic analysis aimed at finding novel proteins that may play a role in regulating neurotransmission - and whose dysfunction may thus lead to a role in neurological disease. Encouraged by these findings, as well as by recent advances in technology, we decided to reinvestigate the trafficking of APP in neurons, using established biochemical techniques in conjunction with new developments in optical methods that allow direct, real-time measurement of protein trafficking in the presynaptic terminal. Here we present data that unambiguously shows endogenous APP to be present at low levels in synaptic vesicles, which undergo stimulation-dependent exo- and endocytosis. Given the number of synaptic vesicles per terminal and the frequency of vesicle cycling, this mode of release can easily account for the majority of APP (derivatives) secreted into the synaptic cleft. Further, we integrate this finding into a revised model for APP trafficking, which fully reconciles the existing data on APP with our current understanding of vesicular release and recycling, in line with the central role of this protein in synaptic function and disease pathology. Finally, we briefly speculate on the implications of our model for the future generation of therapeutics aimed at combating Alzheimer's disease.

## Materials and Methods

Full details of all procedures (including antibodies) can be found in Methods S1.

### Biochemical Procedures

#### Purification of synaptic vesicles

Synaptic vesicles were purified according to standard protocols [14], [15]. Briefly, synaptic vesicles were released from synaptosomes by osmotic lysis, and were further purified by rate-zonal sucrose gradient centrifugation and a final step of size exclusion chromatography on controlled pore glass beads.

#### Mass Spectrometry

Approximately 10–20 µg of synaptic vesicle proteins were separated by 1D SDS-PAGE using a tricine mini-gel [16]. After coomassie staining, lanes were cut into bands and subjected to in-gel trypsinization. Extracted peptides were analyzed by liquid chromatography-coupled MS/MS on an Orbitrap machine (Thermo) and proteins were identified in the National Center for Biotechnology Information (NCBI) non-redundant database using MASCOT software as a search engine [15].

#### Western Blotting

Proteins were separated using a tricine based gel system (as above). Proteins were then transferred to nitrocellulose membrane using standard semi-dry techniques [17]. Membranes were blocked and incubated with primary antibodies overnight at 4°C. HRP-conjugated secondary antibodies were added for one hour at room temperature. Blots were developed using Western Lightning chemiluminescence reagents and images acquired using a CCD reader.

#### Immungold electron microscopy

Electron microscopy was performed as previously described with minor modifications [18]. Synaptic vesicles were absorbed to formvar-coated grids, fixed with 1% paraformaldehyde, quenched with 20 mM glycine and immunostained for synaptophysin and APP. Detection was performed with Protein A-gold. To avoid possible steric masking of APP as a result of synaptophysin staining, a sequential protocol was employed in which APP was labeled first. This was blocked using 1% glutaraldehyde, and then synaptophysin labeling was performed. After counterstaining with 1% uranylacetate, samples were viewed using a CM120 electron microscope, equipped with a TemCam 224A CCD camera.

### Imaging Procedures

#### Cell culture

Hippocampal neurons of the CA3/CA1 region from 1- to 3 day old Wistar rats were prepared in a sparse culture (2,000–5,000 cells per coverslip), and transfected after 3–4 days *in vitro* (DIV) using a modified calcium phosphate transfection procedure. Microscopy was performed at 14–21 DIV [19].

#### 4Pi nanoscopy

Cells were covered with 20 µl of buffer and sealed with a second coverslip coated with sub-resolution red fluorescent beads for focal adjustments (TransFluoSpheres, NeutrAvidinTM labeled microspheres, 0.1 µm diameter; excitation maximum 488 nm, emission maximum 605 nm). The space between the two coverslips was always less than 30 µm. Images were obtained with a commercial 4Pi microscope (Type A-TCS 4Pi, Leica Microsystems).

#### Plasmid constructs

A pHluorin-synaptotagmin-1 vector construct [20] was used to fuse pHluorin N-terminally to an APP cDNA. The APP cDNA (APP695) was obtained by preparation of total RNA from rat brain, subsequent reverse transcription and cDNA amplification by PCR. Primers were designed to include additional recognition sites for Bsu36I and NotI, to allow subsequent cloning of the APP cDNA fragment into the vector after excision of the synaptotagmin-1 cDNA. The sequence of the forward primer was 5′-cctgaggcggatcttccactcgcacac-3′; the reverse primer sequence was 5′-gcggccgcgtcaaaagccgagggtgagtaaat-3′. The integrity of the pHluorin-APP construct was verified by sequencing.

#### Antibody labeling of recycling synaptic vesicles

Antibody labeling was performed with antibodies against synaptotagmin conjugated to cypHer5E. Labeling was performed on pHluorin-APP transfected hippocampal neurons, by incubating the neurons with antibody for 3–4 hours at 37° in a bicarbonate buffer containing (in mM) 120 NaCl, 5 KCl, 1 MgCl_2_, 2.5 CaCl_2_, 10 glucose, 18 NaHCO_3_; pH 7.4 was maintained using 5% atmospheric CO_2_. The cells were then washed twice and placed in a perfusion chamber containing Tyrode solution for imaging.

#### Epifluorescence microscopy of living neurons

Imaging was performed as described previously [20]. A modified Tyrode solution (in mM; 150 NaCl, 4 KCl, 1 MgCl_2_, 2 CaCl_2_, 10 glucose, 10 HEPES buffer, pH 7.4 NaOH) was used for all experiments unless otherwise indicated. Synaptic boutons were stimulated by electric field stimulation (platinum electrodes, 10-mm spacing, 1-ms pulses of 50 mA with alternating polarity). 10 µM CNQX and 50 µM AP5 were added to the bath solution to prevent recurrent synaptic activity as a result of AMPA receptor activation. Fast solution exchanges were achieved using a piezo-controlled stepper device, with a three-barrel glass tubing. The perfusion rate during the experiments was kept at a constant 1 ml/min. To block reacidification of freshly recycled synaptic vesicles, 65 nM folimycin was applied to the neuronal culture before the experiment. For dequenching of vesicular pHAPP, ammonium chloride solution (pH 7.4) was prepared by equimolar substitution of 50 mM NH_4_Cl for NaCl in the Tyrode solution. All other components remained unchanged.

Imaging was performed using a cooled CCD camera mounted on a Zeiss Axiovert 135TV microscope equipped with a 60×, 1.2 NA water-immersion objective and a FITC/Cy5 dual-band filter set. Excitation wavelengths of 480 nm (pHAPP) and 640 nm (cypHer) were produced by a computer-controlled monochromator.

### Ethics statement

All tissues used in this study were obtained from Wistar rats (Charles River, USA), bred and kept for experimental purposes at the Max Planck Institute for Biophysical Chemistry by authorization of the federal state of Lower Saxony, Germany (licence Az. 32.22/Vo). The killing of rats for tissue preparations is not an animal experiment under the terms of federal animal protections laws, so approval by an ethics committee was not necessary. However, all experiments involving the use of animals were carried out following the stringent guidelines issued by the Max Planck Institute, to ensure the highest standards of animal welfare.

## Results

### A routine mass-spectrometry screen detected APP in a preparation of isolated synaptic vesicles

**Figure 1:**
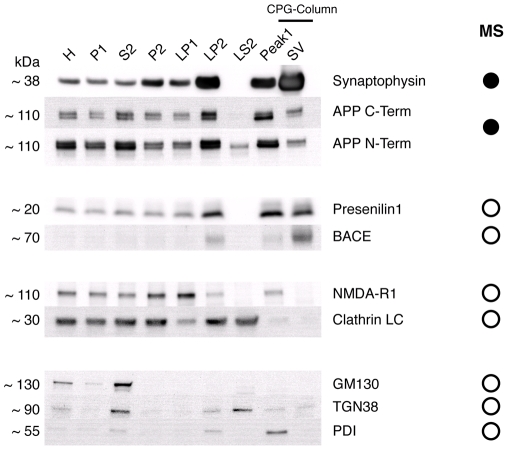
Endogenous full-length APP localizes to isolated synaptic vesicles. APP and its associated processing enzymes were found in a preparation of highly pure synaptic vesicles isolated from rat brain by immunoblotting and mass-spectrometry. Fractions were taken during the preparation of synaptic vesicles by a classical subcellular fractionation protocol. The fractions shown represent increasing levels of purification (left to right): whole brain homogenate (H), large cell fragments and nuclei (P1), crude cytosol - small cell fragments including microsomes, small myelin fragments and soluble proteins (S2), isolated nerve terminals ‘synaptosomes’ (P2), nerve terminal plasma membrane (LP1), crude synaptic vesicles (LP2), presynaptic cytosol (LS2), first peak from the size exclusion column containing larger membranes ∼100–200 nm (Peak1) and highly pure synaptic vesicles (SV). 10 or 20 µg of total protein from each of the individual fractions were subjected to SDS-PAGE followed by immunoblotting. Fully glycosylated APP was routinely found in synaptic vesicles by immunoblotting, as were the APP processing enzymes BACE and Presenilin 1. The neuronal specific isoform of clathrin light chain was undetectable. Contamination by ER-Golgi trafficking vesicles was assessed by immunoblotting for GM130 (cis-Golgi), TGN38 (trans-Golgi network) and protein disulfide isomerase (PDI; Endoplasmic Reticulum). The synaptic vesicle fraction was free of contamination by these proteins. The column MS gives the results obtained from mass-spectrometry using the synaptic vesicle fraction. Proteins found in the SV fraction are indicated with filled circles; proteins not found in the SV fraction are indicated with empty circles.

Although our laboratories recently completed a detailed proteomic analysis of the synaptic vesicle, several known (membrane) proteins were not identified, including the chloride channels ClC3 and ClC7, as well as the vesicular neurotransmitter transporters responsible for monoamine (VMAT) and acetylcholine (VAChT) uptake into vesicles [15]. Difficulties in mass-spectrometry based proteomic analysis usually result from the low abundance of the protein in question, or from the general hydrophobicity of membrane proteins (including the inefficiency of trypsin cleavage). The major strategies currently used to improve peptide sequence coverage include improving the initial fractionation steps (such as protein extraction and the use of different gel types for electrophoresis), analyses using different mass-spectrometer types and repeated analysis of the samples, which together can improve coverage by 20–30% [21]. We decided to reanalyze our synaptic vesicle preparation, in an attempt to find these “missing” proteins, using a new, highly sensitive Orbitrap® machine [22]. For initial screens, we used 1D SDS-PAGE followed by trypsin digestion, as it is generally the most reliable and robust method used to process proteins prior to mass-spectrometry [23]. As predicted, when we searched our peptide list against all entries in the NCBI non-redundant database, we routinely found the most hydrophobic protein in synaptic vesicles (the vesicular ATPase subunit c; as predicted by its Grand Average Hydropathicity (GRAVY) score). Furthermore, we also started to find low abundance proteins such as the vesicular acetylcholine transporter. In addition, we also sequenced a peptide at approximately 100 kDa on the gel that corresponded to amyloid precursor protein (APP). The peptide (ISYGNDALMPSLTETK with an oxidized methionine) maps to amino acids 586–601 of APP. Importantly, this peptide had a Mascot score of 67, indicating that it is highly unlikely that APP was a false-positive identification (see Methods). However, neither of the APP processing enzymes BACE nor Presenilin 1 (a component of the γ-secretase complex) was found by mass-spectrometry (Figure 1).

### Immunoblotting proves that APP is present in highly purified synaptic vesicles

Given that APP is a large protein, containing a large number of potential trypsin cleavage sites and is reasonably hydrophilic (as judged by its GRAVY score), we hypothesized that APP is difficult to detect in synaptic vesicles by mass-spectrometry because it is present in low amounts, consistent with previous studies, which found low levels of APP in rodent brain [11]. Therefore, as an independent means of verification and to compensate, at least in part, for the non-quantitative nature of our proteomic analysis, we monitored the distribution of APP using the complimentary technique of immunoblotting. Analysis of subcellular fractions using our standard marker proteins synaptophysin (an integral membrane protein specific for synaptic vesicles) and the NMDA receptor (a component of the post-synaptic density) revealed that the preparation was enriched in presynaptic synaptic vesicles (Figure 1) [15]. Full-length APP was mostly found in fractions containing endosome-type structures and fragments of plasma membrane as previously reported (fractions S2 and Peak 1 in Figure 1; see legend for further definitions). However, the protein was also found to co-purify at much lower levels with highly pure synaptic vesicles (SV). This result was confirmed using two independent antibodies, specific for either an epitope located at the N- or C-terminal end of the protein; both antibodies showed a similar distribution profile of APP amongst the fractions. The doublet staining found in some fractions reflects the differential glycosylation states of the protein; only fully glycoslyated protein, which is the predominant species found in the synaptic vesicle fraction, is thought to be secreted from the neuron [24]. Importantly, this co-purification pattern was consistently occurring in at least seven full immunoblots, performed using three independent synaptic vesicle preparations. Interestingly, the APP processing enzymes BACE 1 and Presenilin 1 were also routinely found at low levels in the vesicle fraction by immunoblotting; the absence of BACE signal from relatively impure fractions, such as synaptosomes, presumably results from it being present at undetectably low levels, and/or from ‘steric masking’ by tubulin, which is an abundant cytoskeletal protein that runs at the same molecular weight as BACE during SDS-PAGE, but is eventually purified away from synaptic vesicles during the procedure.

The predominant fraction of intracellular APP is thought to be localized to the endoplasmic reticulum, Golgi apparatus and early endosomes; the localization of APP to these biosynthetic organelles being partly explained by the very high rate of synthesis and turnover of this protein (t_1/2_ 1 hr). Immature APP is localized exclusively to the ER, and only mature APP that has been N- and O-glycosylated ever leaves the ER/Golgi compartment(s), via sorting at the TGN [24]. Given the known problems with preparing subcellular fractions free of contamination, it could indeed be that the full-length APP found in the synaptic vesicle fraction actually resulted from low levels of contamination by small membranes originating from these alternate sources. To exclude this possibility, immunoblots for compartment specific markers - GM130 (cis-Golgi), TGN38 (trans-Golgi network) and protein disulfide isomerase (PDI; endoplasmic reticulum) - were performed. None of these proteins were readily detectable in the synaptic vesicle fraction by immunoblotting, nor were they detectable by mass-spectrometry (Figure 1). (See Methods S1 for a list of antibodies and further details).

### Immunoflourescence labeling of neurons shows a fraction of APP localizing to the synaptic vesicle cluster

**Figure 2:**
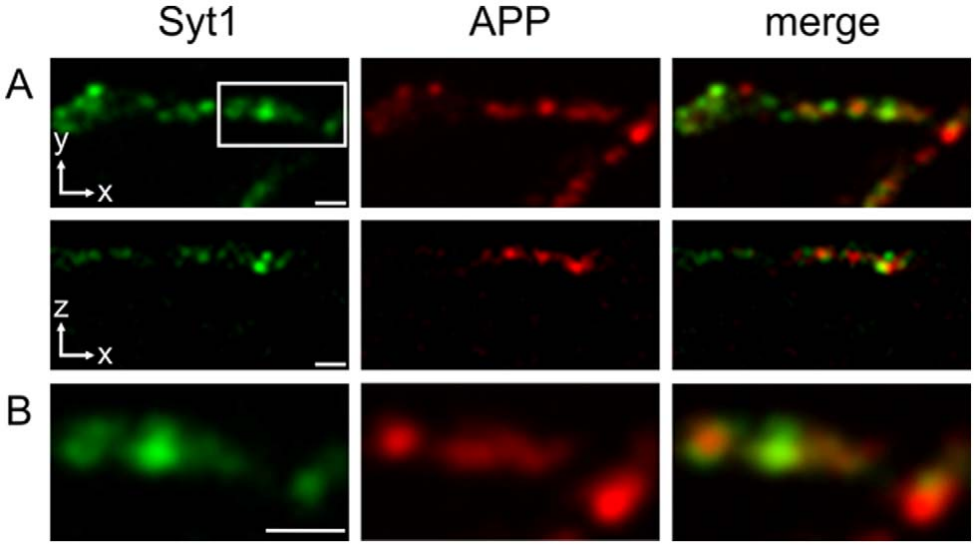
Immunolabeling of fixed hippocampal neurons for APP assessed using 4Pi nanoscopy. Localization of APP to the presynaptic terminal was initially confirmed by double immunofluorescence labeling of cultured hippocampal neurons. Synaptic boutons were detected by immunostaining for the *bona fide* synaptic vesicle protein synaptotagmin 1 (Syt1), using an antibody that binds the N-terminal (luminal) domain of the protein. For APP staining, an antibody directed against the C-terminus of the protein was used. Relative protein distributions were assessed using 4Pi nanoscopy. (A) 4Pi nanoscopy images (upper row: xy- projections, lower row: xz-projections) of neuronal processes stained for synaptotagmin and APP. The APP antibody stained punctate structures that were often elongated in shape, presumably representing APP-transport vesicles. However, some puncta also co-labeled synaptic boutons (see B), as identified by the marker synaptotagmin (coverslips N = 9; synaptic boutons n = 113). (B) Detailed images from (A), corresponding to the area marked by the white box. Synaptotagmin positive presynaptic boutons showed a diffuse APP staining throughout most of the vesicle cluster, suggesting that a small proportion of presynaptic APP is localized to synaptic vesicles. Scale bars 1 µm.

To independently confirm the presynaptic localization of APP we chose to perform immunolabeling on cultured hippocampal neurons, which are an established system for the study of neuronal polarity and structure [20], [25], [26]. Hippocampal neurons were double immunolabeled for APP and the *bona fide* synaptic vesicle marker synaptotagmin 1. Stainings were subsequently imaged using 4 Pi nanoscopy, which provides an improved spatial resolution of approximately 200×200×160 nm (x, y, z) (Figure 2). The APP antibody mainly stained elongated structures that occurred throughout the neurons, and presumably represented APP transport vesicles [27]. However, a fraction of the antibody also labeled synaptic boutons, as judged by co-localization with synaptotagmin 1. Given the increased resolution of the 4Pi nanoscope these structures were unlikely to be large synaptic endosomes, but more likely represent synaptic vesicles containing a small reservoir of APP.

### APP and synaptophysin co-localize on purified synaptic vesicles as revealed by immunogold electron microscopy

**Figure 3:**
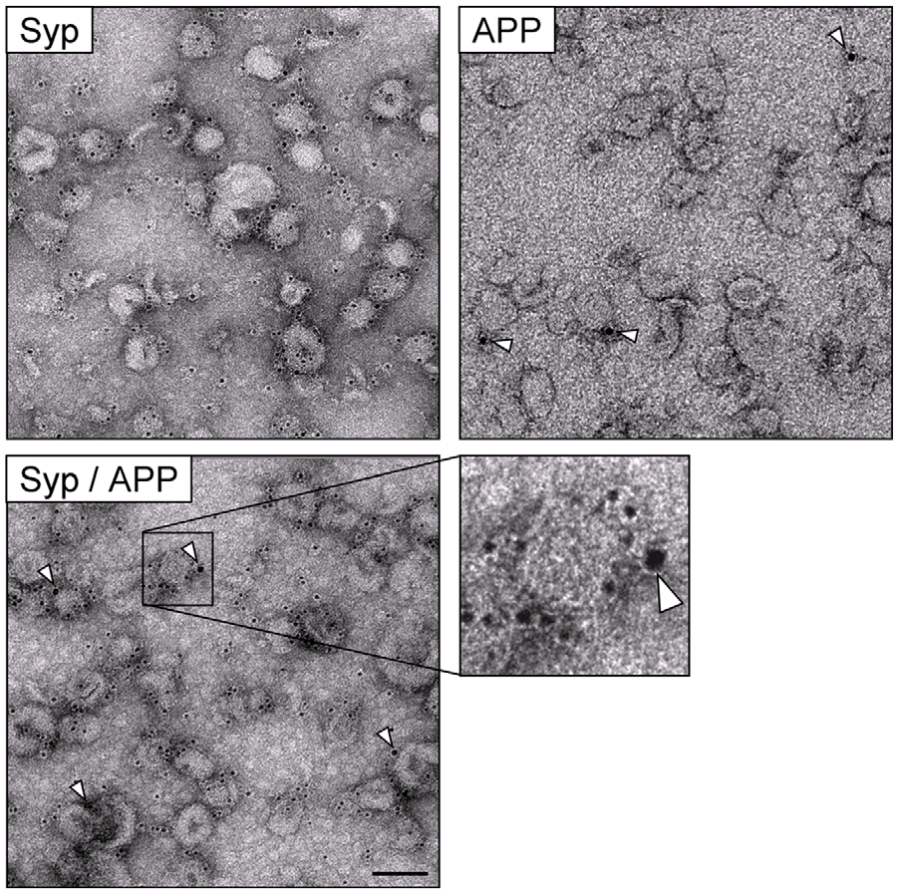
APP and synaptophysin co-localize on a fraction of synaptic vesicles. Synaptic vesicles isolated from rat brain were immunolabeled for synaptophysin (Syp), APP or double labeled (Syp 5 nm gold; APP 10 nm gold), before viewing by negative stain electron microscopy. The low magnification images show the synaptic vesicle preparation to consist solely of small, homogeneously shaped vesicles, with diameters in the range of 40–50 nm. Single labeling resulted in 99% of all vesicles immunopositive for synaptophysin (as previously reported) and 10% of all vesicles immunopositive for APP. Similar results were obtained with double labeling; a double labeled vesicle can clearly be seen in the magnified view. In all images, APP labeling is indicated using arrowheads. For single labeling experiments n = 3; for double labeling experiment n = 2. Scale bars, low magnification 100 nm; high magnification 50 nm.

Although our biochemical and nanoscopy data are in agreement with previous work [12], [13], the techniques themselves are rather indirect. Although laborious, the only method presently capable of visualizing individual synaptic vesicles is electron microscopy. Hence, to directly confirm the localization of APP to synaptic vesicles we performed negative stain immunogold electron microscopy on our isolated synaptic vesicle fraction [18]. Given that the release of processed APP from a synaptic vesicle by exocytosis (either in the form of sAPPα or Aβ) would require the N-terminal end of the protein to be orientated towards the lumen of the vesicle, it is the C-terminal end of the protein that should be most accessible to antibody labeling on the intact vesicle. Hence, immunolabeling was performed with only the C-terminal APP antibody. To overcome potential under-sampling, caused by the low level of APP in synaptic vesicles, many vesicular profiles were counted to ensure relevance (3,677 profiles for APP labeling and 820 profiles for negative control experiments). Figure 3 confirms that the synaptic vesicle preparations used in mass-spectrometry and immunoblotting were of exceptional purity and directly localizes APP to synaptophysin-positive synaptic vesicles (see Figure S1 for controls and single labeling experiments). The preparation consisted solely of small membraneous profiles, with diameters in the range of 40–50 nm [15]. None of the vesicles appeared to be surrounded by electron dense material indicative of a clathrin coat, consistent with our immunoblotting data (Figure 1). The lack of a clathrin coat was confirmed by the fact that antibody labeling would otherwise have been sterically hindered [28]. Approximately, 10% of these vesicles were immunopositive for APP. Similar results were also obtained when synaptic vesicles were double labeled with APP and synaptophysin.

When considering that the vesicle fraction we used for our biochemical analysis represents an ensemble of vesicles isolated from *whole* rat brain, it is possible that our results actually under-represent the number of APP containing vesicles in distinct neuronal regions, such as the hippocampus [29], which are known to be particularly susceptible to amylogenic disease.

### Transiently overexpressed APP traffics to synaptic vesicles: Stimulation dependent exocytosis of pHluorin-APP occurs at synaptic boutons defined by uptake of a cypHer5E-anti-synaptotagmin 1 IgG

Although immunoblotting and mass spectrometry indicate that neuronal clathrin light chain is absent from our synaptic vesicle preparation (Figure 1), we could not definitively exclude that our synaptic vesicle fraction actually contained a small number of endocytic vesicles, which had merely lost their coats during purification [28]. Although clathrin-mediated endocytosis is thought to be the predominant mode of synaptic vesicle recycling in neurons [30] and clathrin coated vesicles are reported to be a major source of vesicular APP [12], it has previously been suggested that vesicle components and APP are separated with 100% efficiency in a sorting intermediate, prior to synaptic vesicle reformation [12]. Although there is increasing evidence that biological trafficking pathways, including synaptic vesicle endocytosis, do not sort protein with 100% efficiency [15], [31], [32], we sought to exclude, as far as possible, that our APP-positive structures were merely ‘de-coated’ vesicles.

We reasoned that *bona fide* synaptic vesicles should undergo stimulation-dependent secretion from neurons. Therefore, we were interested to know how APP-positive vesicles behave under physiological conditions; for instance, do these vesicles undergo exocytosis, or are they refractory to release? Answering this question required the use of an assay that allowed direct visualization of exocytosis in the nerve terminal. For this reason, we again turned to cultured hippocampal neurons, which have been extensively used for the study of synaptic transmission using optical methods [25]. APP constructs (using genetically encoded fluorescent tags) have been extensively reported as mimicking the trafficking of the endogenous protein (including its proteolytic processing), following transient overexpression in cultured neurons [33], [34], [35], [36]. We followed a similar strategy and cloned the 695 amino acid isoform of APP, which is the predominant form found in the nervous system [37], from a rat brain RNA library and attached an N-terminal pHluorin tag to create pHluorin APP (pHAPP). pHluorins are pH sensitive variants of GFP which have been tagged to specific synaptic vesicle proteins and used to quantify synaptic vesicle exo- and endocytosis [25], [38]. Briefly, pHluorin marker systems make use of the fact that the vesicular lumen is acidic – proton transport across the vesicle membrane produces the electrochemical gradient needed for neurotransmitter uptake. In the acidic pH of the vesicular lumen pHluorins are quenched, and only become fluorescent when they are exposed to the more alkaline pH of the external culture media, as a result of exocytosis. The fluorescence signal then recovers following cessation of stimulation as vesicular membranes and proteins are recovered by endocytosis, and synaptic vesicles are reformed and re-acidified. (A schematic of the use of a pHluorin construct in monitoring synaptic activity is given in Figure S2. See also Materials and Methods). As massive overexpression of APP can lead to changes in the APP trafficking pathway, we decided to use the more moderate, neuron-specific synapsin promoter to drive pHAPP expression. Recent work by our group has shown that this promoter only drives the incorporation of 1 or 2 proteins into each vesicle when synaptobrevin 2-pHluorin is transiently overexpressed in hippocampal neurons cultured from a synaptobrevin 2 knock-out animal (despite synaptobrevin 2 being present, on average, at over 60 copies per vesicle in wild-type animals; Sinha et al., submitted). Nevertheless, the overall change in fluorescence recorded during stimulation of neurons expressing synaptobrevin 2-pHluorin is still more than two orders of magnitude greater than that recorded for pHAPP (data not shown), implying that pHAPP is incorporated into only a fraction of vesicles in the synaptic terminal, entirely consistent with our data on endogenous APP sorting (Figures 1, 2, 3).

**Figure 4:**
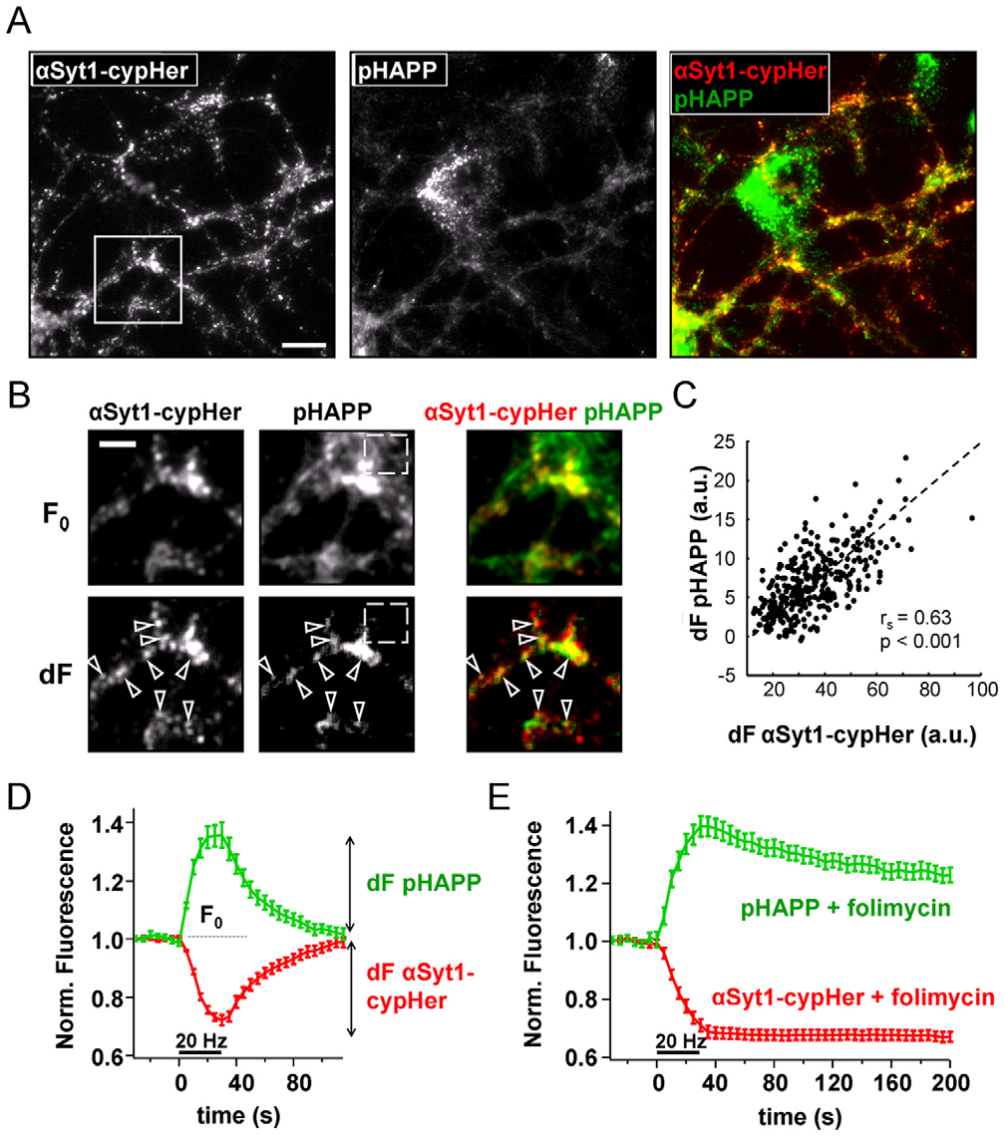
pHluorin-APP and αSyt1-cypHer co-localize at sites of synaptic activity and accurately report synaptic activity. A) Unstimulated neurons showed a punctate staining pattern when labeled with either αSyt1-cypHer or pHAPP. pHAPP staining was typically at the detection limit of the system. Overlaying the images showed that pHAPP partly co-localized with αSyt1-cypHer, which also stained untransfected neurons. Note, consistent with the high rate of synthesis and turnover of APP in neurons, pHAPP also seemed to be concentrated in the Golgi compartment of the neuron. B) Comparison of baseline images (F_0_) and difference images calculated by subtracting the image taken before stimulation from the image taken after stimulation (dF). dF is positive at loci where fluorescence increases upon stimulation (exocytosis) (see also D). As seen in dF images, exocytosis is confined to αSyt1-cypHer positive regions (arrowheads). Images were taken from the region of interest in (A). Stimulation was 30 s, 20 Hz. The boxed region (dashed) in both images illustrates background fluorescence; however, as background fluorescence does not increase upon stimulation it is effectively removed in the difference (dF) image. C) Changes in pHAPP fluorescence (dF) were highly correlated to changes in αSyt1-cypHer fluorescence (arbitrary units). Significance was assessed using a Spearman's rank order test (N = 6; n = 383). D) Time course of fluorescence changes at αSyt1-cypHer positive spots. Synapses labeled with both pHAPP and αSyt1-cypHer show characteristic fluorescence changes upon electrical stimulation (30 s, 20 Hz). Following cessation of the stimulus, fluorescence recovered to pre-stimulus values (average of N = 7 regions, each comprising n>50 boutons; error bars are SEM). E) Folimycin prevented fluorescence recovery at the end of the stimulation. Folimycin is a vacuolar-ATPase inhibitor that blocks the reacidification of synaptic vesicles following endocytosis, proving that pHAPP and αSyt1-cypHer are recovered into synaptic vesicles (average of N = 7 regions, each comprising n>50 boutons; error bars are SEM).

Hippocampal neurons in culture that express pHAPP show very little surface fluorescence (Figure 4), although it is unclear whether this is because pHAPP delivered to the cell surface is efficiently cleaved or endocytosed, or whether pHAPP is exclusively trafficked via an alternative internal pathway. In any case, lack of surface expression made it difficult to correctly identify transfected neurons above background auto-fluorescence (Figures 4A and B). To overcome this problem, we adopted a dual-colour video microscopy approach, in which functional synaptic terminals were co-labeled with a monoclonal antibody against the intra-vesicular domain of the synaptic vesicle protein synaptotagmin 1. Following exocytosis, the intra-vesicular domain of synaptotagmin becomes accessible and can be labeled with an antibody, which is then internalized when the vesicles are retrieved [39]. The monoclonal antibody was coupled to the pH-sensitive Cy-5 dye variant cypHer 5 [40], [41], which can be spectrally separated from pHAPP. Importantly, cypHer fluorescence shows an inverse profile to that of pHluorin, being fluorescent only in the acidic environment of the vesicle following endocytosis (see Figure S2). Together these two markers provide information on both exo- and endocytosis in neurons.

Using this dual labeling strategy, active boutons could be unequivocally identified by the uptake of the cypHer 5 labeled antibody against synaptotagmin1 (αSyt1-cypHer) during vesicle recycling. We thus defined active synaptic boutons as αSyt1-cypHer positive regions and analyzed the corresponding loci in the pHAPP channel. In resting neurons, APP surface fluorescence was co-localized to synaptic boutons labeled with αSyt1-cypHer in only a limited number of regions (Figure 4A). However, when neurons were stimulated electrically (30 s, 20 Hz), pHAPP fluorescence was basically limited to αSyt1-cypHer positive sites (Figure 4B), as judged by the high degree of statistical correlation between the two signals (Spearman's rho = 0.63, p<0.001) (Figure 4C). Importantly, the increase in pHAPP fluorescence was time-locked to the start of stimulation and the overall time course of pHAPP fluorescence mirrored that of αSyt1-cypHer (Figure 4D), suggesting that pHAPP is a synaptic vesicle protein, which cycles in an activity-dependent manner. As an additional control, we also tested whether the decrease of pHAPP fluorescence at the end of stimulation was due to vesicular re-acidification by applying the selective proton pump inhibitor folimycin (N = 6, n>400) [42]. Here the dual marker approach had the additional advantage of confirming the folimycin effect on αSyt1-cypHer kinetics. We found that folimycin inhibited the decrease in pHAPP-fluorescence seen on the cessation of stimulation (Figure 4E) and conclude both that pHAPP traffics to synaptic vesicles and that at least a fraction of the protein is subject to stimulation dependent exocytosis and compensatory endocytosis.

### A fraction of pHluorin-APP is lost from synaptic vesicles during rounds of exo- and endocytosis

**Figure 5:**
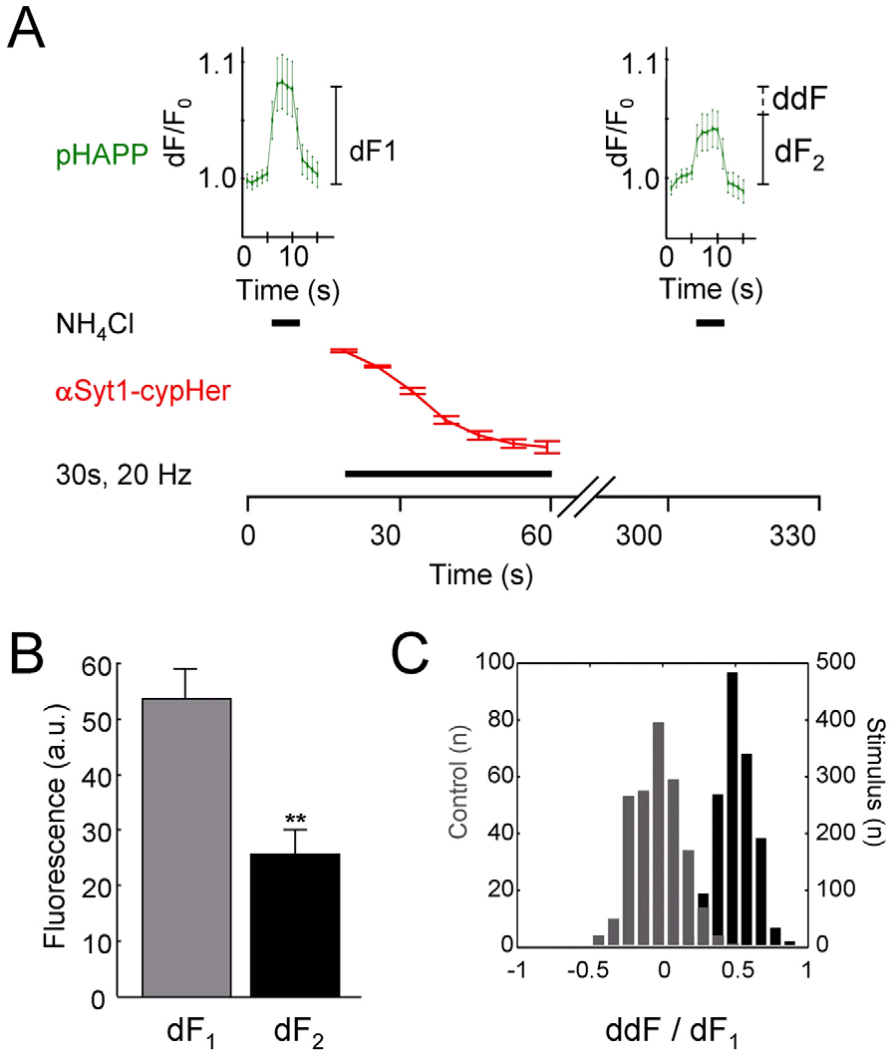
A fraction of pHAPP-fluorescence is lost during electrical stimulation. A) Schematic diagram detailing the time course experiments used to determine total pHAPP at the synapse. pHAPP content (arbitrary flurescence units) was determined before (dF1) and after electrical stimulation of neurons (dF2), by application of NH_4_Cl. Note there was a delay between the cessation of stimulation and application of the second ammonium pulse (illustrated by the broken time axis between 60 s–300 s), to allow completion of endocytosis and reuptake of pHAPP. Loss of pHAPP fluorescence (ddF) was calculated as the absolute difference between dF1 and dF2. To minimize photobleaching, image acquisition in the pHAPP channel was limited to the time during ammonium pulses. Periods of NH_4_Cl addition and electrical stimulation are illustrated by bars on the time axis. αSyt1-cypHer5 fluorescence (arbitrary units) was used as an independent reporter of neuronal activity between the ammonium pulses. Control experiments were performed in an identical fashion, except stimulation was omitted from the protocol. B) Individual boutons show a significant reduction in the absolute NH_4_Cl-induced fluorescence increase following electrical stimulation (30 s, 20 Hz) (p = 0.0013, paired t-test, N = 4, n = 482; t-test performed on N). C) The absolute difference in fluorescence between the two NH_4_Cl pulses (ddF) was normalized by expressing as a function of the total pHAPP in the bouton at the start of the experiment (dF1), to take into account slight differences in expression levels from experiment to experiment. Normalized ddFs obtained under control (grey) and stimulated (black) conditions are plotted as population histograms. Values for control experiments are centered on 0, indicating little, or no overall loss of fluorescence from the terminal. In contrast, stimulation resulted in a shift to 0.5, indicative of fluorescence loss.

While the above data proves that at least a fraction of full-length pHAPP recycles with synaptic vesicles it cannot be discounted that some pHAPP is cleaved during vesicle cycling, particularly considering that the processing enzymes BACE and Presenilin 1 co-purify with synaptic vesicles (Figure 1). If APP was cleaved in the context of exo- and endocytosis, this would result in the loss of the N-terminal fluorescence label on pHAPP following electrical stimulation (Figure S2 A, ‘Cleavage’). Ammonium chloride is membrane permeable and will neutralize the pH of the vesicle lumen; hence, when applied systemically, ammonium chloride will report the entire pHluorin content of the synaptic terminal (Figure S2 A, ‘NH_4_’) [25]. Hence we applied short pulses of ammonium chloride before and after electrical stimulation (45 s, 20 Hz). In order not to induce fluorescence decrease by photobleaching we limited the recordings of pHAPP fluorescence to the ammonium applications (Figure 5A). We found the ammonium-induced pHAPP fluorescence decreased significantly after electrical stimulation (Figures 5B and C). We conclude that the fluorescence loss reflects cleavage of the APP construct C-terminal to the pHluorin tag and subsequent loss into the extracellular medium; in a perfused cell culture system, such as ours, secreted molecules or peptides will be removed in milliseconds from the synaptic cleft. This is entirely consistent with previous studies that have shown correct proteolytic cleavage of APP-GFP constructs [36], and the fact that analysis of pHAPP fluorescence following exocytosis revealed only limited loss through lateral diffusion in the plasma membrane from synaptic sites into adjacent axonal segments (data not shown).

## Discussion

### APP is a *bona fide* synaptic vesicle protein

In this work we reinvestigated the subcellular localization of APP in neurons. Using techniques that allowed direct access to presynaptic mechanisms, we consistently found that small amounts of APP are present in synaptic vesicles, which undergo activity dependent secretion.

At first glance, our findings might seem controversial; over the past decade it has become dogma that APP is absent from synaptic vesicles, and this has heavily influenced the prevailing view of APP trafficking in neurons. However, when we revisited the original literature, we found our results to be entirely consistent with earlier work. In these studies, immunoblotting showed a small fraction of APP was found in isolated synaptic vesicles, and partial co-localization of APP and synaptophysin was demonstrated at the light microscopy level [12], [43]. This was in direct contrast to the large amount found in presynaptic endosomal structures (see below). Given that ‘essential’ trafficking proteins are present in high numbers on all synaptic vesicles (for example, an average rat synaptic vesicle is thought to contain over 60 copies of synaptobrevin 2), it is understandable that the general trend has been to consider only these proteins as *bona fide* functional components [15]. However, both Marquez-Sterling and Ikin emphasized that they could not necessarily exclude APP being a synaptic vesicle component. Our pHluorin-cypHer based experiments now provide strong evidence that APP does actually undergo stimulation-dependent exo- and endocytosis in a small number of synaptic vesicles, explaining the finding that synaptic activity and clathrin-dependent endocytosis are associated with APP trafficking at the synapse [2], [5]. Unfortunately, while our biochemical work also showed APP to be localized to a small subset of synaptic vesicles (and presumably present in low copy number) we were unable to obtain any evidence for APP being localized to distinct *functional* pools of vesicles in the synaptic terminal (although this may also be related to subtle trafficking issues with the construct – see below).

An important remaining question concerns how much APP is actually released by exocytosis of synaptic vesicles. Previously, it was thought that the Aβ-peptides formed from APP processing were secreted from exosomes in the neuron. However, this form of release was found to account for only 1% of the total peptide secretion [44]. Thus, while there is evidence that these organelles are secreted by neuronal activity, their overall number and contribution to release is minor when compared to synaptic vesicle turnover [45]. Given the number of synaptic vesicles per terminal and the frequency of vesicle cycling [46] we think it is likely that this mode of release actually accounts for the vast majority of APP (and its derivatives) secreted into the synaptic cleft - potentially providing the missing link between synaptic activity and extracellular Aβ levels.

### A revised model of APP trafficking at the synapse

**Figure 6:**
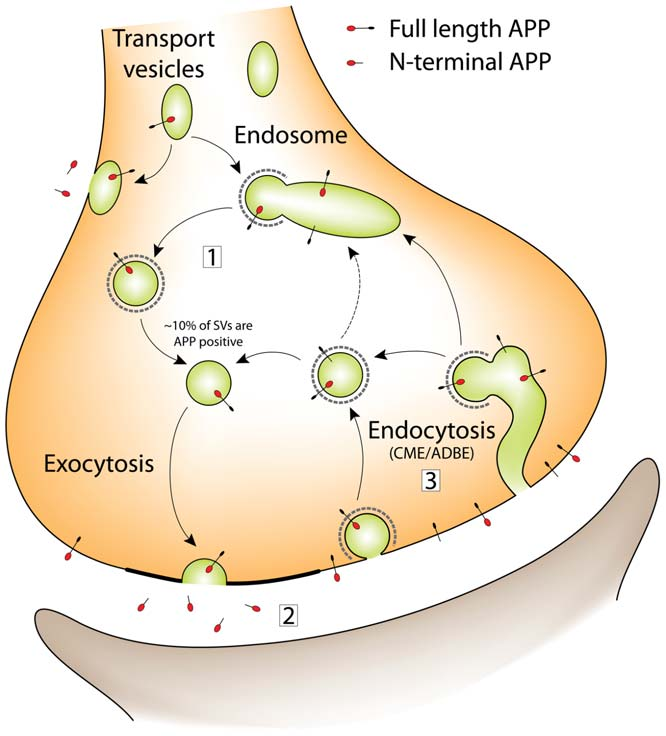
A revised model for APP trafficking in the presynaptic terminal. The figure illustrates the various recycling pathways proposed for synaptic vesicles in the presynaptic terminal, and how APP recycling can be integrated. Synaptic vesicle precursors are brought to the presynaptic terminal in transport vesicles. It is thought that these transport vesicles undergo a round of fusion with the plasma membrane followed by retrieval and sorting, possibly in an endosomal intermediate, to form fully functional synaptic vesicles, which are capable of undergoing fusion with the plasma membrane. Following exocytosis, vesicles are retrieved from the plasma membrane by endocytosis. Under physiological conditions this is thought to occur via a clathrin-mediated endocytosis (CME) pathway; it is still unclear whether vesicles lose their coats and are recycled directly, or whether they pass through an endosomal sorting intermediate. Putative endosomes may in fact be formed by activity dependend bulk endocytosis (ADBE) of plasma membrane, which is thought to occur during periods of heavy stimulation. APP trafficking at the synapse can be integrated into our current understanding of synaptic vesicle recycling. It is known that APP is also delivered to the presynaptic terminal in transport vesicles. These transport vesicles either fuse with the plasma membrane, depositing APP on the plasma membrane surface, or alternatively they fuse with an endosomal sorting intermediate (which we postulate is identical to that used during recycling of synaptic vesicles). Hence, synaptic vesicles could incorporate APP when recycling through the endosome (1). During synaptic vesicle exocytosis, APP cleavage products would then be released (2). Slight infidelities in the endocytic process might also mean small amounts of surface-resident APP could be endocytosed, along with *bona fide* synaptic vesicle proteins. These vesicles may then recycle directly for subsequent rounds of fusion and APP release, or pass through the endosomal system (3). Alternatively APP may be internalized and recycled into vesicles as a result of bulk endocytosis.

Identifying the synaptic vesicle as a secretory organelle for APP closes the trafficking cycle for this protein at the synapse. In Figure 6 we propose a revised trafficking model, based on that originally put forward by Cirrito and colleagues [2], which fully reconciles our new findings to existing work - both on APP trafficking and general synaptic physiology.

Our schematic attempts to illustrate more clearly the similarities and interconnectedness of the trafficking pathways. In particular, we propose that the presynaptic endosome plays a crucial role in linking synaptic vesicle and APP cycling.

Synaptic vesicles may recycle through one of several pathways in the presynaptic terminal. Synaptic vesicle precursors are brought to the presynaptic terminal in transport vesicles. It is thought that these transport vesicles undergo a round of fusion with the plasma membrane followed by retrieval and sorting, possibly in an endosomal intermediate to form fully functional synaptic vesicles [47]. These vesicles are then competent to undergo Ca^2+^ mediated fusion with the plasma membrane, in response to neuronal stimulation. Following exocytosis, vesicles are retrieved from the plasma membrane by endocytosis. Under physiological conditions this is thought to occur via a clathrin-mediated [30], and presumably dynamin-dependent [48], pathway. It is still unclear whether vesicles then lose their coats and are recycled directly (blurring the traditional distinction between a ‘clathrin-coated’ and ‘synaptic’ vesicle), or whether they pass through an endosomal sorting intermediate from which vesicles are reformed using similar dynamin and clathrin-dependent mechanisms [49]. Alternatively, it is possible that both these pathways operate in parallel with a small proportion of vesicles being recycled through endosomes as part of a ‘quality control mechanism’ to ensure correct protein and lipid sorting [32]; explaining not only why a small proportion of actively recycling vesicles contain the endosomal markers Rab5, syntaxin 13, syxtaxin 6 and vti1a [15], [32], but also why non-quantitative immunodepletion of synaptic vesicles from brain extract, using general vesicle markers such as synaptobrevin, may fail to detect proteins present either in low copy number or localized to discrete vesicular subsets [12], [43]. Interestingly, putative endosomes may even be initially formed by bulk endocytosis of plasma membrane, which is thought to occur during periods of heavy stimulation; and given the stimulus paradigms that we used in our study at least some contribution of this bulk-endocytosis is likely [50].

APP is also thought to be trafficked to the presynaptic terminal in transport vesicles, which interestingly seem to contain *bona fide* synaptic vesicle components such as Rab3a and synaptobrevin 2 [27]. These transport vesicles are thought to either fuse with the plasma membrane, depositing APP on the plasma membrane surface [2], or fuse directly with an endosomal sorting intermediate (which we postulate is identical to that used during recycling of synaptic vesicles) [2], [51]. Hence, synaptic vesicles could acquire APP at two points in this recycling pathway. Synaptic vesicles could incorporate APP when recycling through the endosome (step 1); and during subsequent rounds of exocytosis APP cleavage products would be released (step 2). Alternatively, at the plasma membrane, slight infidelities in the endocytic process might lead to small amounts of surface-resident APP being endocytosed, along with *bona fide* synaptic vesicle proteins [32]. These vesicles could then be recycled directly for subsequent rounds of fusion and APP release, or pass through the endosomal system (step 3). Alternatively, surface APP may be efficiently internalized and recycled into vesicles as a result of activity dependent bulk endocytosis (ADBE).

Obviously, APP needs to be proteolytically processed at some point during trafficking. In this respect, it is interesting that our data shows pHAPP fluorescence decreased significantly after neuronal stimulation, consistent with proteolytic cleavage of the construct (although we were unable to determine the exact nature of the cleavage products due to limitations in our optical tools). One possible explanation is cleavage of APP at the plasma membrane surface by α-secretase. An alternative explanation, however, is that APP is processed to Aβ in synaptic vesicles, a proposal which is supported by complementary data from Frkyman and colleagues who recently found Aβ using biochemical methods in a somewhat cruder preparation of isolated synaptic vesicles [13]. In this respect, it is interesting that we also found BACE and Presenilin 1 (β and γ secretases) by immunoblotting in our synaptic vesicle fractions (although as yet we have been unable to detect either of these proteins using mass-spectrometry - presumably reflecting the difficulty of detecting low copy number proteins using this technique [21]). Interestingly, this finding implicates at least a proportion of APP positive vesicles as recycling through the endosome; APP and BACE are conveyed to the synaptic terminal in distinct transport vesicles [36] and hence require such an obligate sorting intermediate. Importantly, BACE is maximally active at pH 5.0–5.5 [52], which is close to the luminal pH of synaptic vesicles (pH 5.7) [38]. There is also growing evidence that cholesterol- and sphingolipid-rich membrane microdomains are involved in regulating trafficking and processing of APP, by organising the protein and its processing enzymes into discrete domains [53], [54], consistent with the high cholesterol content of synaptic vesicles (40 mol%) [15]. Given that independent studies show Aβ production to be dependent on alkalization (such as occurs during synaptic vesicle exocytosis) [55], [56], as well as dynamin dependent endocytosis [2], it is tempting to speculate on the presence of a regulated, proteolytic processing complex in synaptic vesicles. Such a complex may be regulated by the action of the protein Reticulon 3, which was also found in our synaptic vesicle fraction (data not shown), and is known to inhibit the activity of BACE [57]. The presence of a regulated processing complex may go some way to explaining why a fraction of pHAPP remains intact and is endocytosed during our experiments; although the possibility that our construct artificially drives expression of pHAPP to vesicles which do not participate in APP processing and release under normal physiological conditions cannot be completely discounted. We are currently investigating these aspects of APP trafficking and release.

### Implications for synaptic function

Conceptually, exocytic release of APP (derivatives) from synaptic vesicles is an attractive possibility; modulation of synaptic function, particularly by Aβ, would be possible over a time course of seconds to minutes, in direct response to alterations in neuronal activity. Furthermore, it appears that APP does not have to be specifically enriched in synaptic vesicles to achieve such effects. There is increasing evidence that even picomolar concentrations of low n-number oligomers (particularly trimers) of Aβ rapidly and effectively inhibit NMDA receptor activity, leading to reduced Ca^2+^ influx into the dendritic spine, with subsequent spine shrinkage and retraction leading to an overall reduction in neuronal spine density. These effects promote long-term depression (LTD), inhibiting the induction of long-term potentiation (LTP) by NMDA receptor-dependent signaling. As LTP is thought to be the neural correlate of learning and memory, this would explain why Aβ can produce memory impairment when overproduced [58].

The synaptic depressing effects of Aβ are interesting when coupled to findings indicating that increased neural activity can drive the processing of APP to Aβ [59]. These two findings led Manilow and colleagues to suggest a negative feedback system that could function to scale neuronal output during periods of intense activity; the effects of such a system could be successfully localized to discrete points of high neuronal activity by limiting the amount of Aβ released, and maximising the spatial sampling of the dendritic spine [60]. In this model, high levels of neural activity drive formation and release of small amounts of Aβ, which then depresses synaptic transmission reducing neural activity. Thus, an Aβ-mediated negative feedback system could be regarded as a homeostatic process that becomes dysregulated in Alzheimer's disease [7], explaining why increased synaptic activity causes a rapid and sustained increase in Aβ, with brain regions that show the highest default activity being most at risk of developing AD, while reducing synaptic activity lowers Aβ load [5]. Recognizing that oligomer toxicity leads to synaptic dysfunction, which presumably precedes plaque formation, also provides an explanation for the observation that subtle brain dysfunction can be detected in certain individuals many years before the appearance of the senile plaques thought to coincide with the onset of Alzheimer's disease.

In the future, it will be interesting to delineate the exact mechanisms which couple synaptic vesicle recycling with APP trafficking, processing and secretion – with a view to developing more effective therapeutic strategies. Indeed, pharmacologic modulation of synaptic transmission [61] and cognitive training [62] are already established strategies to slow down the progress of Alzheimer's disease. Identifying the synaptic vesicle as the principle organelle of APP trafficking in the synapse, however, raises the hope of developing more subtle treatments that selectively modulate protein processing and release.

## Supporting Information

Figure S1 **APP is localized to synaptic vesicles as shown by immunogold electron microscopy.** Top; synaptic vesicles isolated from rat brain were immunolabeled for synaptophysin, APP or double labeled (synaptophysin 5 nm gold; APP 10 nm gold), before viewing by negative stain electron microscopy. The low magnification images show the synaptic vesicle preparation to consist solely of small, homogeneously shaped vesicles, with diameters in the range of 40–50 nm. The insets show higher magnification images of vesicles from the same field of view. Single labeling resulted in 99% of all vesicles immunopositive for synaptophysin (as previously reported) and 10% of all vesicles immunopositive for APP. Similar results were obtained with double labeling. Bottom; negative control experiments in which the primary antibody was omitted. For single labeling experiments n = 3; for double labeling experiment n = 2. Scale bars, low magnification 100 nm; high magnification 50 nm.
(TIF)

Figure S2 **The use of pH sensitive probes to monitor exo- and endocytosis.** A) Schematic illustrating the design of the pHAPP construct and its use in monitoring synaptic activity. pHluorins are pH sensitive variants of GFP which can be tagged to specific synaptic vesicle proteins and used to quantify synaptic vesicle exo- and endocytosis. When pHluorin is attached N-terminally to APP, it is directed towards the lumen of the synaptic terminal, which is acidic under resting conditions. Thus the pHluorin will be quenched (‘Initial situation’). During neuronal stimulation, synaptic vesicles undergo fusion with the plasma membrane, and the luminal surface becomes exposed to the more alkaline pH of the external culture media, and the fluorescence of the pHluorin increases (‘Exocytosis’). Following exocytosis the fluorescence signal is reduced, either due to compensatory endocytosis (vesicle reformation and re-acidification; ‘Endocytosis’), or from loss of the N-terminal tag into the culture media as a result of proteolytic processing (‘Cleavage’). Ammonium chloride is membrane permeable and will neutralize the pH of the vesicle lumen; hence, ammonium chloride can be used to report the entire pHluorin content of the synaptic terminal (‘NH_4_’). B) Schematic illustrating the use of αSyt1-cypHer antibodies to monitor synaptic activity. Following exocytosis, the intravesicular domain of synaptotagmin 1 is exposed to the external culture media and can be labeled with an antibody, which is internalized when the vesicles are retrieved. This antibody is directly conjugated to the dye cypHer 5. CypHer fluorescence shows an inverse profile to that of pHluorin, being fluorescent only in the acidic environment of the vesicle (‘Initial situation’). Following exocytosis, cypHer fluorescence is quenched in the alkaline pH of the culture media (‘Exocytosis’). Following endocytosis, cypHer fluorescence increases as the reformed vesicle is reacidified.
(TIF)

Methods S1 (DOC)
