# Supplementary figures and images for: Amyloid Precursor Protein Is Trafficked and Secreted via Synaptic Vesicles
Source: PLoS One. 2011 Apr 27;6(4):e18754. doi: 10.1371/journal.pone.0018754 (PMC3083403; doi:10.1371/journal.pone.0018754)

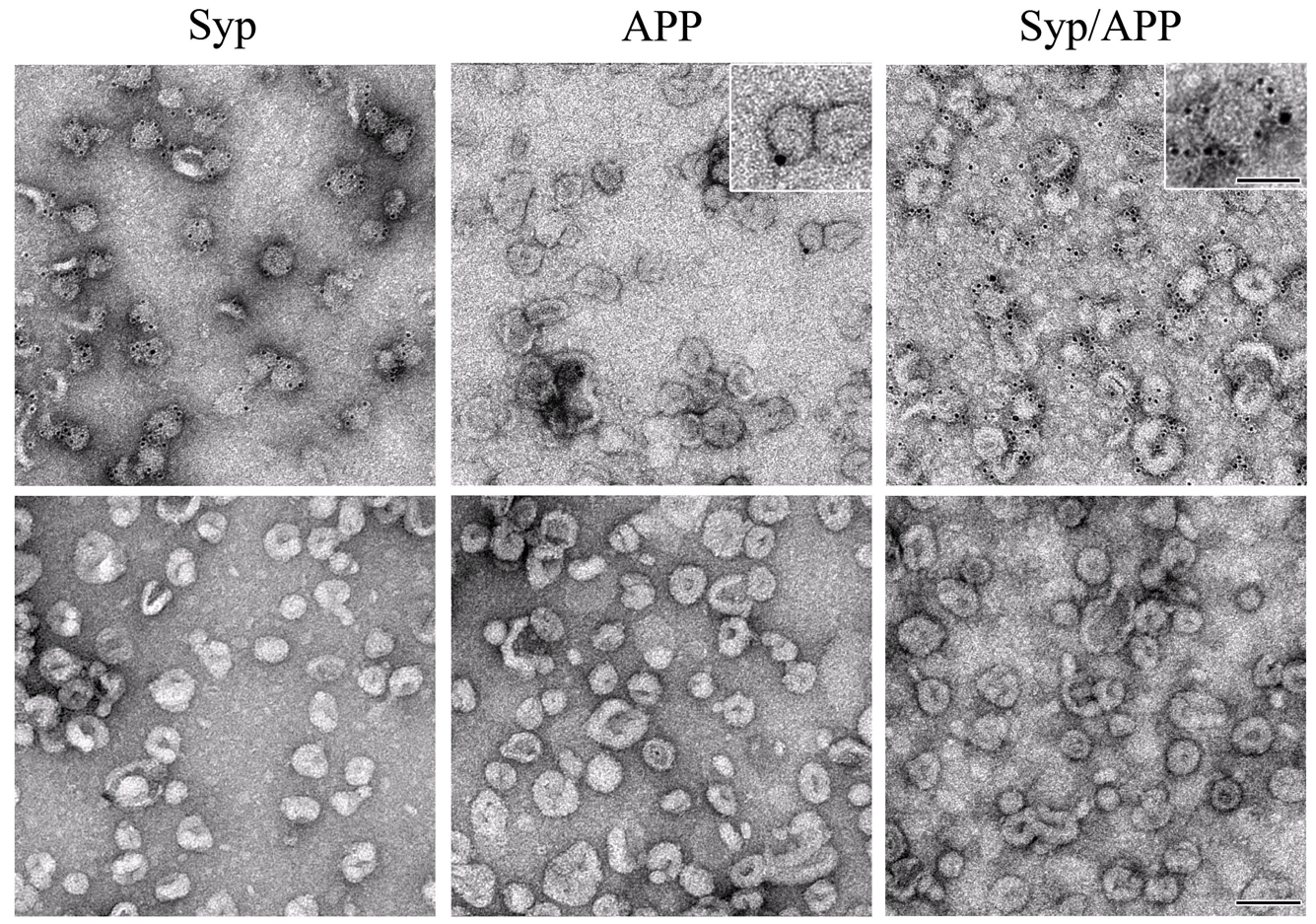

Supplement: Figure S1 — APP is localized to synaptic vesicles as shown by immunogold electron microscopy. Top; synaptic vesicles isolated from rat brain were immunolabeled for synaptophysin, APP or double labeled (synaptophysin 5 nm gold; APP 10 nm gold), before viewing by negative stain electron microscopy. The low magnification images show the synaptic vesicle preparation to consist solely of small, homogeneously shaped vesicles, with diameters in the range of 40–50 nm. The insets show higher magnification images of vesicles from the same field of view. Single labeling resulted in 99% of all vesicles immunopositive for synaptophysin (as previously reported) and 10% of all vesicles immunopositive for APP. Similar results were obtained with double labeling. Bottom; negative control experiments in which the primary antibody was omitted. For single labeling experiments n = 3; for double labeling experiment n = 2. Scale bars, low magnification 100 nm; high magnification 50 nm. (TIF) [file pone.0018754.s001.tif]

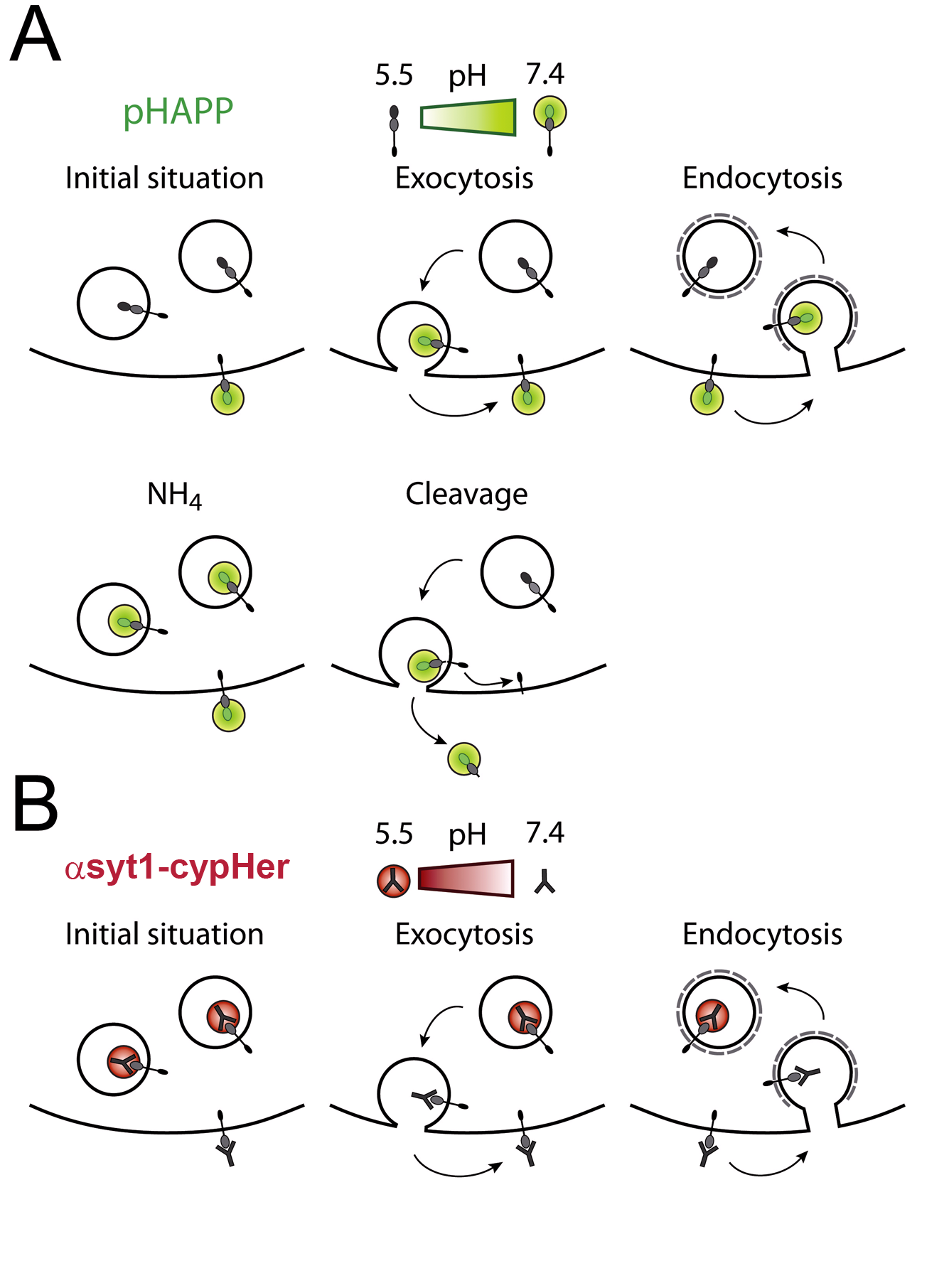

Supplement: Figure S2 — The use of pH sensitive probes to monitor exo- and endocytosis. A) Schematic illustrating the design of the pHAPP construct and its use in monitoring synaptic activity. pHluorins are pH sensitive variants of GFP which can be tagged to specific synaptic vesicle proteins and used to quantify synaptic vesicle exo- and endocytosis. When pHluorin is attached N-terminally to APP, it is directed towards the lumen of the synaptic terminal, which is acidic under resting conditions. Thus the pHluorin will be quenched (‘Initial situation’). During neuronal stimulation, synaptic vesicles undergo fusion with the plasma membrane, and the luminal surface becomes exposed to the more alkaline pH of the external culture media, and the fluorescence of the pHluorin increases (‘Exocytosis’). Following exocytosis the fluorescence signal is reduced, either due to compensatory endocytosis (vesicle reformation and re-acidification; ‘Endocytosis’), or from loss of the N-terminal tag into the culture media as a result of proteolytic processing (‘Cleavage’). Ammonium chloride is membrane permeable and will neutralize the pH of the vesicle lumen; hence, ammonium chloride can be used to report the entire pHluorin content of the synaptic terminal (‘NH4’). B) Schematic illustrating the use of αSyt1-cypHer antibodies to monitor synaptic activity. Following exocytosis, the intravesicular domain of synaptotagmin 1 is exposed to the external culture media and can be labeled with an antibody, which is internalized when the vesicles are retrieved. This antibody is directly conjugated to the dye cypHer 5. CypHer fluorescence shows an inverse profile to that of pHluorin, being fluorescent only in the acidic environment of the vesicle (‘Initial situation’). Following exocytosis, cypHer fluorescence is quenched in the alkaline pH of the culture media (‘Exocytosis’). Following endocytosis, cypHer fluorescence increases as the reformed vesicle is reacidified. (TIF) [file pone.0018754.s002.tif]
