# Supplementary material for: Amyloid Precursor Protein Is Trafficked and Secreted via Synaptic Vesicles
Source: PLoS One. 2011 Apr 27;6(4):e18754. doi: 10.1371/journal.pone.0018754 (PMC3083403; doi:10.1371/journal.pone.0018754)
Supplement: Methods S1 — (DOC) [file pone.0018754.s003.doc]

**Supporting Information Methods**

**Biochemical Procedures**

**Purification of synaptic vesicles**

Subcellular fractionation of rat brain, allowing the preparation of highly purified synaptic vesicles, was performed as described [1]. According to this protocol, a crude synaptosomal fraction was first isolated by differential centrifugation. The aim of this primary subfractionation was to reduce contamination from small vesicles found in the cell bodies of neurons (such as those associated with the endoplasmic reticulum). The synaptosomes were then lysed by osmotic shock and synaptic vesicles released into the medium. After removal of synaptosomal fragments and larger membranes from the lysate, synaptic vesicles were sedimented using high-speed centrifugation. The resulting pellet, already five to sixfold enriched in synaptic vesicles, was then further purified by rate zonal ultracentrifugation on a sucrose gradient, followed by size-exclusion chromatography on controlled pore glass beads. Synaptic vesicles prepared using this method are of the highest purity, being morphologically homogeneous as judged by electron microscopy, with more than 99% of them carrying major synaptic vesicle proteins, such as synaptophysin and synaptobrevin 2.

Protein determination was performed using a modified Lowry assay [2]. When necessary, protein precipitation was performed using a chloroform:methanol based protocol [3].

**Mass spectrometry**

Approximately 10-20 µg of synaptic vesicle proteins were separated by 1D SDS-PAGE using a standard 10% tricine mini-gel [4]. After Colloidal Coomassie Blue staining, lanes were cut into bands and subjected to in-gel trypsinization. The extracted peptides were analyzed by liquid chromatography-coupled tandem MS (liquid chromatography MS/MS) on an LTQ Orbitrap XL machine (Thermo) and proteins were identified in the National Center for Biotechnology Information (NCBI) non-redundant database using MASCOT 2.2 software (Matrix Science) as a search engine [1], with a mass accuracy of ± 5 ppm for the parent ion (MS) and ± 0.8 Da for the fragment ions (MS/MS). Searches were constrained to 2 missed cleavages, while carbamidomethylation of cysteines and oxidation of methionine residues were considered variable modifications. Peptides with a peptide score ≥ 20 were considered for further analysis. A full description of statistical methods used for peptide validation (including the Mascot/Peptide score) can be found at the Matrix Science website – www.matrixscience.com.

Other protein analysis tools used were ProtPram, Peptide Cutter and SIM-Align, which are freely available at www.expasy.org.

**Western blotting**

Proteins were separated using a tricine based gel system, with 8%, 10% or 16% acrylamide in the resolving gel to achieve maximal separation in the desired size range (as above). Proteins were transferred to nitrocellulose membrane using standard semi-dry techniques [5]. Transfer time was increased when using higher percentage acrylamide gels to ensure sufficient transfer. Nitrocelluose membranes were stained with Ponceau S and gels with Coomassie Blue to verify efficient transfer. Membranes were then cut in the appropriate size range using prestained molecular weight marker as a guide (Fermentas). Primary antibody incubations were performed overnight at 4°C. A complete list of primary antibodies can be found in Table S1. After washing, blots were incubated with HRP-conjugated secondary antibodies for one hour at room temperature. Blots were then developed using Western Lightning enhanced chemiluminescence reagents (Perkin Elmer) and a LAS CCD reader (Fuji). Images were exported to Adobe Photoshop PS1 (Adobe) and were contrast enhanced using the ‘Auto Levels’ function to avoid pixel saturation.

**Immunogold electron microscopy**

Immunogold electron microscopy was performed as previously described with minor modifications [6]. Purified synaptic vesicles were absorbed to formvar-coated grids, fixed with 1% paraformaldehyde, quenched with 20 mM glycine and immunostained using a polyclonal antibody against synaptophysin (G95) or a monoclonal antibody against APP (Synaptic Systems), followed by addition of Protein A-gold (10 nm). The preparations were then washed repeatedly with Tris-bufffered saline (TBS, containing in mM; 150 NaCl, 50 Tris, pH7.4 KOH) and high-salt TBS (500 NaCl) and post-fixed with 2% glutaraldehyde. To avoid the possible steric masking of APP (low copy number synaptic vesicle protein) as a result of synaptophysin staining (high copy number protein), a sequential protocol was employed for double staining, in which APP was labeled first (10 nm Protein-A gold grains). This immunoreaction was then blocked by fixation with 1% glutaraldehyde, followed by the second immunogold labeling for synaptophysin (5 nm Protein-A gold), as described above. As a negative control, primary antibodies were omitted from incubations. After counterstaining with 1% uranylacetate, samples were viewed using a CM120 Philips electron microscope, equipped with a TemCam 224A slow scan CCD camera (TVIPS). Quantification was performed as follows; the total number of immunolabeled particles on each grid was counted and then expressed as a percentage of the total number of vesicles present. Figures were corrected for background staining.

A full list of all antibodies used in the study can be found in Table S1.

**Imaging Procedures**

**Cell culture**

Primary cultures of rat hippocampal neurons were prepared from the CA3/CA1 region of 1 or 2 day-old Wistar rats as previously described [7] and transfected at 3 days *in vitro* (DIV) by a modified calcium phosphate transfection procedure [8]. For 4Pi nanoscopy rat hippocampal neurons from 1-3 day old Wistar rats were prepared and seeded on glass coverslips according to the protocol published by Delgado-Martinez et al. [9]. All imaging experiments were carried out at 14-28 DIV at room temperature.

**Immunocytochemistry**

For immunocytochemistry, hippocampal neurons were grown directly on glass coverslips, using standard culture techniques. Labeling steps were all carried out at room temperature. Culture media was removed and neurons were washed three times in staining buffer (in mM; 120 NaCl, 5 KCl, 3.5 MgCl_2_, 10 glucose, 1 EGTA, 10 HEPES; pH 7.40 NaOH), before fixation in 4% paraformaldehyde for 10 min. Paraformaldehyde was then removed by washing the cells with staining buffer (four washes each of 3 min duration), before permeabilizing with staining buffer supplemented with 2% goat serum, 1% bovine serum albumin (BSA) and 0.4% saponin for 30 min. Primary antibodies against APP and synaptotagmin 1 were then added in staining buffer containing 2% goat serum and 1% BSA and left for 60 min. After 4 washes, neurons were incubated with secondary antibodies (goat anti-mouse Alexa 488 and goat anti-rabbit Alexa 594) in buffer containing 2% goat serum and 1% BSA for 1 h at room temperature. Coverslips were then washed again 4 times, before mounting and using for 4 Pi nanoscopy.

**4Pi nanoscopy**

Cells were covered with 20 µl of buffer and sealed with a second coverslip coated with sub-resolution red fluorescent beads (TransFluoSpheres®, NeutrAvidinTM labeled microspheres, 0.1 µm: excitation maximum 488 nm; emission maximum 605 nm). The space between the two coverslips was always less than 30 µm. Images were obtained with a commercial 4Pi nanoscope (Type A-TCS 4Pi, Leica Microsystems), using water immersion lenses (63x, NA 1.2). For two photon excitation, a mode-locked Ti:sapphire laser (MaiTai, Spectra Physics GmbH, Germany) with the pulse length stretched to 1.2 ps was used. The laser was tuned using a grating to a wavelength between 790 and 820 nm. The beam expander was set to 3. Fluorescence originating from the sample was passed through a filter cube (SP700, BS560, BP500–550, and BP607–683), and its intensity was measured using photon counting avalanche photodiodes (Perkin Elmer). The detection pinhole was set between 0.72 and 0.86 Airy units. Samples were mounted between the two microscope objectives, and the focus and phase of the counter-propagating beams were prealigned to the immobilized beads. Then xz-stacks of the cells were recorded using a pixel size between 14 x 14 nm and 19 x 19 nm in the xz -direction and a step size of 97 nm in the y-direction. 4Pi raw images were then brightened, rescaled and subsequently filtered using the ‘Gaussian Blur’ function found in the image-processing program ImageJ (National Institutes of Health, USA). A detailed description of 4Pi analysis has been published recently [10]. Briefly, the intensity profile of the main maximum of the point spread function (PSF) in both of the two detection channels was independently fitted to an elliptical 2D gaussian function, using a commercial plug-in for ImageJ (ILTracker, Ingo Lepper Software/Consulting, Germany) and the distance between the two centers was calculated. Due to the improved resolution in the z direction, only distances in this direction were used in our analysis.

For three-dimensional (3D) reconstructions ‘ghost images’, which arise in 4Pi nanoscopy because of side lobes from the PSF, were eliminated by deconvolution using Leica software, which is based on a linear three-point or five-point deconvolution. The reconstructions were then derived from image stacks using the Leica software.

**Plasmid constructs**

A pHluorin-synaptotagmin-1 vector construct [11] was used to fuse pHluorin N-terminally to an APP cDNA. The APP cDNA (APP695) was obtained by preparation of total RNA from rat brain, subsequent reverse transcription and cDNA amplification by PCR. Primers were designed to include additional recognition sites for Bsu36I and NotI, to allow subsequent cloning of the APP cDNA fragment into the vector after excision of the synaptotagmin-1 cDNA. The sequence of the forward primer was 5´-cctgaggcggatcttccactcgcacac-3´; the reverse primer sequence was 5´-gcggccgcgtcaaaagccgagggtgagtaaat-3´. The integrity of the pHluorin-APP construct was verified by sequencing.

**Antibody labeling of recycling synaptic vesicles**

The validity of using pHluorins to monitor synaptic vesicle exo- and endocytosis has recently been questioned, as the behaviour of the various fusion proteins can vary considerably [12]; for instance, following exocytosis, a proportion of synaptobrevin 2-pHluorin diffuses through the plasma membrane and is not recovered during endocytosis [11], whereas synaptophysin-pHluorin remains relatively clustered and is fully endocytosed [13]. At the moment, it is still a matter of debate as to whether this reflects the genuine behaviour of the individual synaptic protein(s), or if the process of tagging the protein influences its behaviour. To circumvent any possible problem, we also employed an independent marker system – simultaneous antibody labeling of a synaptic vesicle protein [14]. Thus, we are confident that pHAPP undergoes exocytosis and endocytosis in synaptic vesicles. Further, although pHAPP fluorescence is lost to a considerable degree during electrical stimulation, unlike other pHluorin-constructs, we believe that this is in agreement with the independent finding of APP cleavage products in crude synaptic vesicles using biochemical methods (as discussed in the main text).

Antibody labeling was performed with antibodies against synaptotagmin1 conjugated to cypHer5E (Synaptic Systems). Labeling was performed on pHAPP transfected hippocampal neurons, by incubating the neurons with antibody for 3-4 hours at 37° in a bicarbonate buffer containing (in mM) 120 NaCl, 5 KCl, 1 MgCl_2_, 2.5 CaCl_2_, 10 glucose, 18 NaHCO_3_; pH 7.4 was maintained using 5% atmospheric CO_2_. The cells were then washed twice and placed in a perfusion chamber containing Tyrode solution for imaging.

**Epifluorescence microscopy of living neurons**

Imaging was performed essentially as described previously [11]. A modified Tyrode solution (in mM; 150 NaCl, 4 KCl, 1 MgCl_2_, 2 CaCl_2_, 10 glucose, 10 HEPES buffer, pH 7.4 NaOH) was used for all experiments unless otherwise indicated. Synaptic boutons were stimulated by electric field stimulation (platinum electrodes, 10-mm spacing, 1-ms pulses of 50 mA with alternating polarity). 10 µM 6-cyano-7-nitroquinoxaline-2,3-dione (CNQX) and 50 µM D,L-2-amino-5-phosphonovaleric acid (AP5) were added to the bath solution to prevent recurrent synaptic activity as a result of AMPA receptor activation. Fast solution exchanges were achieved using a piezo-controlled stepper device (SF77B, Warner Instruments), with a three-barrel glass tubing. The perfusion rate during the experiments was kept at a constant 1 ml/min. To block reacidification of freshly recycled synaptic vesicles, 65 nM folimycin was applied to the neuronal culture before the experiment. For dequenching of vesicular pHAPP, ammonium chloride solution (pH 7.4) was prepared by equimolar substitution of 50 mM NH_4_Cl for NaCl in the Tyrode solution. All other components remained unchanged.

Imaging was performed using a cooled slow-scan CCD camera (SensiCam-QE, PCO) mounted on an inverted microscope (Axiovert 135TV, Zeiss) equipped with a 60x, 1.2 NA water-immersion objective (C-Apochromat, Zeiss) and an FITC/Cy5 dual-band filter set (AHF). Excitation wavelengths of 480 nm (pHAPP) and 640 nm (cypHer) were produced by a computer-controlled monochromator (Polychrom V, Till Photonics). In order to minimize photobleaching of the fluorescent labels acquisition rates were adjusted to 0.2 Hz and 2s for both pHAPP and CypHer channels. Images were also recorded in a ‘stacked’ manner – αSyt1-cypHer followed by pHAPP.

To avoid introducing any bias into the analysis through the manual selection of functional synaptic terminals, an automated detection algorithm was used. Three images before and after stimulation were averaged and a difference image was built from the average images. Peaks were detected in the difference image using automatic spot-detection of synaptic bouton size regions [15]. All image and data analysis was performed using custom-written routines in MATLAB (The MathWorks Inc.). Paired t-test, Spearman’s rank correlation coefficient and one-sample Kolmogorov-Smirnov (KS) test were computed using built-in routines in MATLAB.

| Protein | Supplier | Reference |
| --- | --- | --- |
| Synaptophysin | Custom Polyclonal | [16] |
| Synaptophysin | SySy - Cat. No. 101 011 | [16] |
| Synaptotagmin 1 | SySy – Cat. No. 105 221 | [14] |
| Synaptotagmin 1 | SySy – Cat. No. 105 311CpH* | [14] |
| NMDA Receptor Subunit | SySy - Cat. No. 114 011 | [17] |
| Clathrin Light Chain | SySy – Cat. No. 113 001 | [18] |
| APP N-Terminal | Millipore – Cat. No. MAB348 | [19] |
| APP C-Terminal | SySy - Cat. No. 127 003 | [20] |
| Presenilin 1 | Millipore – Cat. No. MAB5232 | [21] |
| BACE | Millipore – Cat. No. MAB5308 | [22] |
| GM130 | BD – Cat. No. 610823 | [23] |
| TGN38 | BD – Cat. No. 610899 | [24] |
| PDI | BD – Cat. No. 610947 | [25] |

**Table 1. A list of immunological reagents used in this study.**

*Cypher dye is conjugated directly to the synaptotagmin antibody.

All antibodies were used according to the supplier’s instructions.

SySy; Synaptic Systems (www.sysy.com). Millipore (www.millipore.com). BD; BD Transduction Laboratories (www.bdbiosciences.com).

For western blot detection, HRP-conjugated secondary antibodies were purchased from Bio-Rad (www.bio-rad.com). For immunofluorescence, fluorescently labeled secondary antibodies were obtained from Invitrogen (www.invitrogen.com).

**References**

1. Takamori S, Holt M, Stenius K, Lemke EA, Grønborg M, et al. (2006) Molecular anatomy of a trafficking organelle. Cell 127: 831-846.

2. Peterson GL (1977) A simplification of the protein assay method of Lowry et al. which is more generally applicable. Anal Biochem 83: 346-356.

3. Wessel D, Flügge UI (1984) A method for the quantitative recovery of protein in dilute solution in the presence of detergents and lipids. Anal Biochem 138: 141-143.

4. Schägger H (2006) Tricine-SDS-PAGE. Nat Protoc 1: 16-22.

5. Towbin H, Staehelin T, Gordon J (1979) Electrophoretic transfer of proteins from polyacrylamide gels to nitrocellulose sheets: procedure and some applications. Proc Natl Acad Sci U S A 76: 4350-4354.

6. Takamori S, Riedel D, Jahn R (2000) Immunoisolation of GABA-specific synaptic vesicles defines a functionally distinct subset of synaptic vesicles. J Neurosci 20: 4904-4911.

7. Liu G, Tsien RW (1995) Synaptic transmission at single visualized hippocampal boutons. Neuropharmacology 34: 1407-1421.

8. Threadgill R, Bobb K, Ghosh A (1997) Regulation of dendritic growth and remodeling by Rho, Rac, and Cdc42. Neuron 19: 625-634.

9. Delgado-Martinez I, Nehring RB, Sørensen JB (2007) Differential abilities of SNAP-25 homologs to support neuronal function. J Neurosci 27: 9380-9391.

10. Hüve J, Wesselmann R, Kahms M, Peters R (2008) 4Pi microscopy of the nuclear pore complex. Biophys J 95: 877-885.

11. Wienisch M, Klingauf J (2006) Vesicular proteins exocytosed and subsequently retrieved by compensatory endocytosis are nonidentical. Nat Neurosci 9: 1019-1027.

12. Opazo F, Punge A, Bückers J, Hoopmann P, Kastrup L, et al. (2010) Limited intermixing of synaptic vesicle components upon vesicle recycling. Traffic 11: 800-812.

13. Granseth B, Odermatt B, Royle SJ, Lagnado L (2006) Clathrin-mediated endocytosis is the dominant mechanism of vesicle retrieval at hippocampal synapses. Neuron 51: 773-786.

14. Kraszewski K, Mundigl O, Daniell L, Verderio C, Matteoli M, et al. (1995) Synaptic vesicle dynamics in living cultured hippocampal neurons visualized with CY3-conjugated antibodies directed against the lumenal domain of synaptotagmin. J Neurosci 15: 4328-4342.

15. Sbalzarini IF, Koumoutsakos P (2005) Feature point tracking and trajectory analysis for video imaging in cell biology. J Struct Biol 151: 182-195.

16. Jahn R, Schiebler W, Ouimet C, Greengard P (1985) A 38,000-dalton membrane protein (p38) present in synaptic vesicles. Proc Natl Acad Sci U S A 82: 4137-4141.

17. Jockusch WJ, Speidel D, Sigler A, Sørensen JB, Varoqueaux F, et al. (2007) CAPS-1 and CAPS-2 are essential synaptic vesicle priming proteins. Cell 131: 796-808.

18. Ferguson SM, Brasnjo G, Hayashi M, Wölfel M, Collesi C, et al. (2007) A selective activity-dependent requirement for dynamin 1 in synaptic vesicle endocytosis. Science 316: 570-574.

19. Loers G, Aboul-Enein F, Bartsch U, Lassmann H, Schachner M (2004) Comparison of myelin, axon, lipid, and immunopathology in the central nervous system of differentially myelin-compromised mutant mice: a morphological and biochemical study. Mol Cell Neurosci 27: 175-189.

20. Christensen DZ, Bayer TA, Wirths O (2009) Formic acid is essential for immunohistochemical detection of aggregated intraneuronal Abeta peptides in mouse models of Alzheimer's disease. Brain Res 1301: 116-125.

21. Zhou S, Zhou H, Walian PJ, Jap BK (2005) CD147 is a regulatory subunit of the gamma-secretase complex in Alzheimer's disease amyloid beta-peptide production. Proc Natl Acad Sci U S A 102: 7499-7504.

22. Kametaka S, Shibata M, Moroe K, Kanamori S, Ohsawa Y, et al. (2003) Identification of phospholipid scramblase 1 as a novel interacting molecule with beta -secretase (beta -site amyloid precursor protein (APP) cleaving enzyme (BACE)). J Biol Chem 278: 15239-15245.

23. Ireton RC, Davis MA, van Hengel J, Mariner DJ, Barnes K, et al. (2002) A novel role for p120 catenin in E-cadherin function. J Cell Biol 159: 465-476.

24. Ozawa K, Kondo T, Hori O, Kitao Y, Stern DM, et al. (2001) Expression of the oxygen-regulated protein ORP150 accelerates wound healing by modulating intracellular VEGF transport. J Clin Invest 108: 41-50.

25. Jenne N, Frey K, Brügger B, Wieland FT (2002) Oligomeric state and stoichiometry of p24 proteins in the early secretory pathway. J Biol Chem 277: 46504-46511.
